# Single *cis*‐elements in brassinosteroid‐induced upregulated genes are insufficient to recruit both redox states of the BIL1/BZR1 DNA‐binding domain

**DOI:** 10.1002/1873-3468.70147

**Published:** 2025-08-29

**Authors:** Shohei Nosaki, Masae Ohtsuka, Takeshi Nakano, Masaru Tanokura, Takuya Miyakawa

**Affiliations:** ^1^ Institute of Life and Environmental Sciences University of Tsukuba Japan; ^2^ Tsukuba Plant‐Innovation Research Center (T‐PIRC) University of Tsukuba Japan; ^3^ Department of Applied Biological Chemistry, Graduate School of Agricultural and Life Sciences The University of Tokyo Japan; ^4^ Graduate School of Biostudies Kyoto University Japan

**Keywords:** BIL1, brassinosteroid, BZR1, DNA‐binding domain, H_2_O_2_, PIF4, transcription factor

## Abstract

The plant‐specific transcription factor BRZ‐INSENSITIVE‐LONG 1 (BIL1)/BRASSINAZOLE‐RESISTANT 1 (BZR1) regulates the growth of *Arabidopsis thaliana* via brassinosteroid signaling, acting as both a gene repressor and activator. Its upregulation requires environmental cues mediated by PHYTOCHROME INTERACTING FACTOR 4 (PIF4), with oxidative modifications via H_2_O_2_ enhancing their interaction. However, the nature of the tripartite complex of *cis*‐elements, BIL1/BZR1, and PIF4 under redox changes remains unclear. Here, we demonstrate that oxidation of the DNA‐binding domain (DBD) of BIL1/BZR1 alters its DNA‐binding ability. However, single *cis*‐elements enriched in brassinosteroid‐induced genes do not support binding of either redox form, nor does BIL1/BZR1 DBD heterodimerize with PIF4 DBD on these elements. These findings highlight the complexity of brassinosteroid transcriptional regulation beyond DNA‐binding specificity and redox modifications.

## Graphical abstract

Phytohormone brassinosteroid‐induced gene regulation by the transcription factor BIL1/BZR1 involves redox‐dependent DNA‐binding alternation and interaction with the transcription factor PIF4. The reduced BIL1/BZR1 dimer binds preferred *cis*‐elements, while oxidation alters its oligomerization state and disrupts DNA‐binding ability. Single *cis*‐elements are insufficient for regulation, implying cooperation with additional elements, cofactors, or disordered regions. This model explains the complexity of brassinosteroid‐responsive transcriptional control.

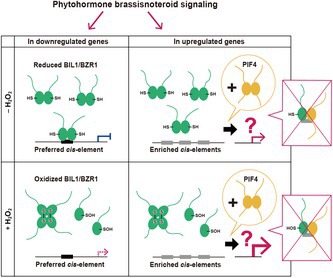

## Abbreviations

BBS BIL1/BZR1‐binding sequence

BIAM biotinylated iodoacetamide

BIL1 BRZ‐INSENSITIVE‐LONG 1

BR brassinosteroid

BRRE brassinosteroid‐response element

BZR1 BRASSINAZOLE‐RESISTANT 1

bHLH basic helix–loop–helix

CBB Coomassie Brilliant Blue

DBD DNA‐binding domain

DTT dithiothreitol

FAM carboxyfluorescein

MBP maltose‐binding protein

mMBP mutated maltose‐binding protein

PAE predicted aligned error

PDB Protein Data Bank

pLDDT predicted local distance difference test

PIF4 PHYTOCHROME INTERACTING FACTOR 4

TF transcription factor

**Fig. 1:**
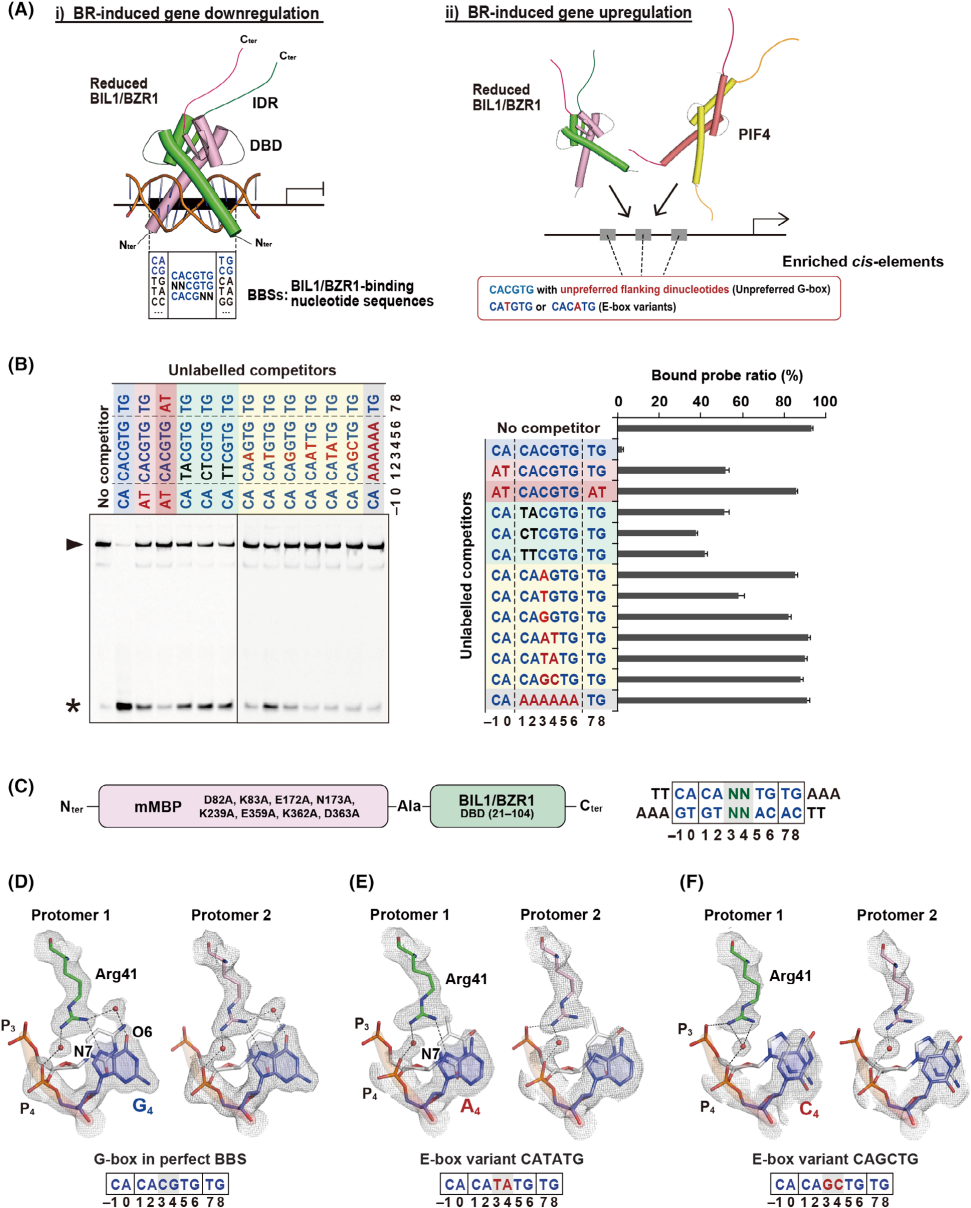
DNA‐binding property of the reduced BIL1/BZR1 DBD. (A) Proposed mechanism of the dual gene regulation by BIL1/BZR1. (i) Downregulation of BR‐induced genes: BIL1/BZR1 forms a homodimer that preferentially recognizes 10‐bp BIL1/BZR1‐binding sequences, termed ‘BBSs’, which include the G‐box (CACGTG) and BRRE (CGTG^C^/_T_G). This strong interaction facilitates gene downregulation in cooperation with a specific corepressor. (ii) Upregulation of BR‐induced genes: BIL1/BZR1 acts as a coactivator with PIF4 to enhance gene expression. This upregulation is possibly associated with the enrichment of sequences such as the ‘unpreferred G‐box’ flanked by specific dinucleotides and the E‐box variant CATGTG (complementary to CACATG) in the target genes. (B) Competition experiments based on gel shift assay were conducted using 4 μm of the reduced MBP‐fused BIL1/BZR1 DBD and 0.25 μm of a FAM‐labeled fragment containing CA|CACGTG|TG, one of the perfect BBSs, mixed with 50‐fold excess of unlabeled DNA fragments as competitors. The nucleotide sequences of unlabeled competitors are shown. The nucleobases replaced from CA|CACGTG|TG are indicated in red letters. The electrophoretic patterns are shown in the left panel with asterisks (*) and triangular arrowheads (▲) indicating the position of the free FAM‐labeled DNA probe and MBP‐fused BIL1/BZR1‐DNA complex, respectively. The bar graph in the right panel shows the fluorescence densitometric profile. Data are the means ± SEM of three independent experiments. (C) Constructs for cocrystallization of mMBP‐fused BIL1/BZR1 DBD and E‐box‐containing DNA fragments. (D–F) Structural comparison of the interaction with Arg41 and the nucleobase at position 4 (N_4_ base) between the G‐box motif in the perfect BBS (C_3_G_4_) (PDB: 5ZD4^14^) (D) and two variants of the E‐box motif, T_3_A_4_ (E) and G_3_C_4_ (F), under accession codes of 9 M73 and 9 M74, respectively. Electron density maps of Arg41 and the N_4_ base are shown as a 2*F*_o_–*F*_c_ map contoured at 1.0σ. Water molecules mediating hydrogen bonding networks are shown as red spheres. Hydrogen bonds are depicted as black dashed lines. Protomers 1 and 2 of the reduced BIL1/BZR1 DBD correspond to chains C and D, respectively, in the assembly with a higher *B*‐factor across all three structures deposited in the PDB^14^.

The plant‐specific transcription factor (TF) BRZ‐INSENSITIVE‐LONG 1 (BIL1)/BRASSINAZOLE‐RESISTANT 1 (BZR1) and its homologs regulate a wide range of biological processes including plant growth and development, primarily via phytohormone brassinosteroid (BR) signaling [1, 2, 3, 4, 5, 6]. In *Arabidopsis thaliana*, BR‐activated BIL1/BZR1 affects hundreds of genes, upregulating some and downregulating others [7, 8]. Initially, BIL1/BZR1 had been proposed to recognize the brassinosteroid‐response element (BRRE; CGTG^C^/_T_G) and the E‐box motif (CANNTG), especially the G‐box motif (CACGTG) [5, 6, 7, 8]. Our recent study identified 10‐bp *cis*‐elements enriched in the promoters of BR‐induced downregulated genes [9], which we hereby designate as BIL1/BZR1‐binding sequences (BBSs) (Fig. 1A). Among these, the C^G^/_A_|CACGTG|^C^/_T_G elements, also containing both G‐box and BRRE, most strongly recruit BIL1/BZR1, and are therefore regarded as ‘perfect’ BBSs. In contrast, promoters of upregulated genes predominantly contain CACGTG with unpreferred flanking dinucleotides, such as the unpreferred G‐box or the E‐box variant CATGTG (complementary to CACATG) [7, 8, 10] (Fig. 1A).

A previous study suggested that BIL1/BZR1 functions as a coactivator of PHYTOCHROME INTERACTING FACTOR 4 (PIF4), a member of the basic helix–loop–helix (bHLH) family TFs from *A. thalian*a [10]. PIF4 is a crucial regulator that integrates light and temperature signals to modulate plant growth and development [11, 12]. Since the DNA‐binding domain (DBD) of BIL1/BZR1 possesses an amino acid motif similar to those of typical bHLH family members, including PIF4 (Fig. 1A), it has been proposed that BIL1/BZR1 and PIF4 might heterodimerize through their bHLH‐type DBDs to activate target genes [6, 10, 13]. Our recent structural analysis revealed that the BIL1/BZR1 DBD adopts a dimerization conformation distinct from those of the bHLH family TFs [14]. Notably, BIL1/BZR1 features conserved cysteine residues within its DBD that are reactive to hydrogen peroxide (H_2_O_2_)‐mediated signals [15, 16, 17], which are associated with both stress responses and growth in plants [15, 18, 19, 20]. The oxidation of these cysteine residues has been suggested to facilitate the interaction between BIL1/BZR1 and PIF4, thereby enhancing gene activation [20]. However, the direct observation of the tripartite complex involving known *cis‐*elements, BIL1/BZR1, and PIF4, under either redox state, has yet to be achieved. Consequently, the molecular mechanism underlying the crosstalk between BR, light/temperature, and stress signaling remains poorly understood. In this study, we aimed to update and integrate a model of gene regulation involving BIL1/BZR1 through H_2_O_2_‐mediated oxidation and its interaction with PIF4, focusing on their DBDs, which have been proposed as key domains for the interaction between BIL1/BZR1 and PIF4.

## Materials and methods

### Protein preparation

The mutated maltose‐binding protein (mMBP)‐Ala‐BIL1/BZR1 DBD (amino acids 21–104) protein for crystallization, along with the MBP‐fused BIL1/BZR1 DBD (21–104) and unfused BIL1/BZR1 DBD (21–104), as well as the MBP‐fused PIF4 DBD (253–319) for biochemical assays, were expressed and purified as described previously [9, 14, 21]. All protein samples underwent buffer exchange into a reductant‐free buffer (20 mm Tris/HCl, pH 8.0, 1 M NaCl, 10% glycerol) via size exclusion chromatography (SEC). This was performed using a Superdex 200 10/300 GL Increase column (Cytiva, Tokyo, Japan) or a Superdex 75 10/300 GL Increase column (Cytiva) operated on an ÄKTA go protein purification system (Cytiva). The purified proteins were reconcentrated, and their concentrations were determined by measuring absorbance at 280 nm with a NanoDrop One spectrophotometer (Thermo Fisher Scientific, Waltham, MA, USA).

### Oxidation treatment

The unfused BIL1/BZR1 DBD (21–104) at a concentration of 0.5 mm was treated with an oxidant‐containing buffer (20 mm Tris/HCl, pH 8.0, 1 M NaCl, 10% glycerol, 1 mm H_2_O_2_) on ice for 16 h. The oxidant‐treated samples, in a volume of 300 μL, were then loaded onto the Superdex 75 10/300 GL Increase column using the ÄKTA go system, with the same reductant‐free buffer for SEC analysis. The eluted samples were subsequently separated by SDS/PAGE using an ATTO Mini Size Polyacrylamide Gel Electrophoresis system (ATTO Corporation, Tokyo, Japan) and stained with Coomassie Brilliant Blue (CBB). To estimate the apparent molecular mass (MM), the following standards were used from the Gel Filtration Standard kit #1511901 (Bio‐Rad Laboratories, Hercules, CA, USA): bovine globulin (MM 158 kDa), chicken ovalbumin (MM 44 kDa), and equine myoglobin (MM 17 kDa).

### Crystallization and structure determination

The mMBP‐Ala‐BIL1/BZR1 DBD (21–104) proteins and DNA fragments containing the E‐box variants (CATATG or CAGCTG) were cocrystallized using methods previously described with some modifications [9, 14]. Crystals of the BIL1/BZR1‐DNA complexes were obtained using the sitting drop vapor diffusion technique, with a reservoir solution consisting of 50 mm sodium cacodylate (pH 6.5), 0.2 M potassium chloride, 0.01 M magnesium chloride, and 10% (w/v) polyethylene glycol 4000. The setup was maintained at 293 K. All crystals were subsequently transferred to a reservoir solution supplemented with 26% ethylene glycol as a cryoprotectant and were flash‐cooled to 95 K, with annealing performed. X‐ray diffraction data were collected at beamline AR‐NE3A at the Photon Factory in Tsukuba, Japan, using a Pilatus‐2 M detector. The data were processed with XDS [22, 23, 24] and scaled by AIMLESS [25]. Molecular replacement was conducted using MOLREP from the CCP4 suite [26, 27], employing the crystal structure of mMBP‐Ala‐BIL1/BZR1 (21–104) in complex with the G‐box‐containing DNA fragment (PDB ID: 5ZD4) as a template model. Refinement cycles were performed using PHENIX [28] and COOT [29]. Data collection and refinement statistics are detailed in Table 1. Structural visualizations were created using the PyMOL viewer (Version 2.5; Schrödinger, LLC, New York, NY, USA). Overall *B*‐factors and root mean square deviation (RMSD) values for structural comparisons were also calculated using PyMOL.

**Table 1:** X‐ray data collection and refinement statistics of mMBP‐BIL1/BZR1 (21–104) in complex with DNA containing E‐box variants.

	E‐box (T_3_A_4_)	E‐box (G_3_C_4_)
Data collection		
Space group	*P*2_1_	*P*2_1_
Cell dimensions		
*a*, *b*, *c* (Å)	102.9, 92.6, 111.8	102.8, 92.5, 111.7
β (°)	100.3	100.2
Resolution (Å)	46.30–2.31 (2.35–2.31)*	47.27–2.23 (2.27–2.23)
*R*_meas_	0.090 (1.05)	0.086 (1.061)
*R*_pim_	0.047 (0.557)	0.045 (0.568)
<*I*/σ(*I*)>	16.0 (2.1)	16.7 (2.1)
Unique reflections	90 599 (4450)	100 538 (4938)
Completeness (%)	100.0 (100.0)	100.0 (97.8)
Redundancy	7.0 (6.9)	6.9 (6.8)
CC(1/2)	0.999 (0.746)	0.999 (0.705)
Refinement		
Resolution (Å)	43.62–2.31	43.58–2.23
Unique reflections	90 505	100 452
*R*/*R*_free_	0.205/0.245	0.215/0.244
No. of atoms		
Protein	13 584	13 592
DNA	1220	1220
Solvent	219	215
Ligand (maltose)	92	92
*B*‐factors (Å^2^)		
Protein	58.7	54.6
DNA	68.7	62.7
Solvent	42.9	39.5
Ligand (maltose)	39.6	38.0
RMS deviations		
Bond lengths (Å)	0.006	0.005
Bond angles (°)	0.692	0.662
Ramachandran plot (%)		
Favored region	98.0	98.0
Allowed region	2.0	2.0
Outliers	0.0	0.0

* Values in parentheses are for the highest‐resolution shell.

### Gel shift assay

Carboxyfluorescein (FAM)‐labeled and nonlabeled DNA fragments were prepared, and competition experiments using the MBP‐fused BIL1/BZR1 DBD (21–104) were performed as described previously [9]. On the other hand, for experiments using the unfused BIL1/BZR1 DBD or the MBP‐fused PIF4 DBD, 1 μm of either DBD was mixed with 0.25 μm of labeled DNA fragments and 50 ng·μL^−1^ poly(dI‐dC)·poly(dI‐dC) in a reaction buffer consisting of 10 mm Tris/HCl (pH 7.5), 50 mm KCl, 50 mm NaCl, and either 0 or 5 mm dithiothreitol (DTT), supplemented with 2.5% glycerol. Subsequently, 10 μL of each mixture was loaded onto a 10% polyacrylamide gel in 0.5 × TBE buffer (45 mm Tris/HCl, pH 8.2, 45 mm boric acid, 1 mm EDTA). Fluorescence on the gel was detected using the preset settings for Epi‐blue excitation at 466 nm and a BPF535 bandpass filter at 535 nm, utilizing a LuminoGraph III WSE‐6300 imaging system (ATTO Corporation).

### Amino acid sequence alignment

For amino acid sequence alignment, the following proteins were used: *A. thaliana* BIL1/BZR1 (At1g75080; UniProt ID: Q8S307), BES1 (At1g19350; UniProt ID: Q9LN63), BEH1 (At3g50750; UniProt ID: Q9S7F3), BEH2 (At4g36780; UniProt ID: Q94A43), BEH3 (At4g18890; UniProt ID: O49404), and BEH4 (At1g78700; UniProt ID: Q9ZV88); *Nicotiana tabacum* LOC107792445 (UniProt ID: A0A1S4A0Q1); *Solanum lycopersicum* 101 246 895 (UniProt ID: A0A3Q7FF78); *Glycine max* 100 803 869 (UniProt ID: I1K7U0); and *Oryza sativa* 07 g0580500 (UniProt ID: Q7XI96). CLUSTAL OMEGA [24] was used for multiple sequence alignments using default parameters, and the results were displayed by ESPript 3.0 [25].

### Structure prediction

AlphaFold3 predictions [30] were carried out via the AlphaFold server (https://alphafoldserver.com). For the structural modeling of the DNA‐BZR1‐PIF4 complexes, specific segments of BZR1 DBD (amino acids 21–104) and PIF4 DBD (amino acids 253–319) were used, along with a 16 bp double‐stranded DNA fragment containing an unpreferred G‐box (TC|CACGTG|TC). The generated model was evaluated using the predicted local distance difference test (pLDDT) scores and the predicted aligned error (PAE) values. The highest‐ranked structure was visualized as a cartoon model using PyMOL (version 2.5; Schrodinger, LLC).

## Results

### The reduced BIL1/BZR1 does not preferentially recognize the E‐box elements

Certain *cis‐*elements containing the E‐box motif variant CATGTG, which are common in upregulated genes, have been identified as targets of BIL1/BZR1 either *in vitro* or *in vivo*. However, the relative differences in their BIL1/BZR1‐binding capabilities have not been thoroughly investigated. To address this, we employed a reduced form of BIL1/BZR1 containing only the DBD, which was fused to MBP. We tested its interaction with various nucleobase variants based on the sequence C_−1_A_0_|C_1_A_2_C_3_G_4_T_5_G_6_|T_7_G_8_, which is a canonical perfect BBS. The reduced BIL1/BZR1 showed stronger binding to the perfect BBS than to its variants, consistent with previous findings [9] (Fig. 1B). Replacing C_−1_A_0_ with A_−1_ T_0_ on one side weakened its BIL1/BZR1‐binding ability such that it was equal to or even lower than that of C_1_A_2_ with other bases, including T_1_A_2_, C_1_T_2_, and T_1_T_2_, while replacing C_−1_A_0_|T_7_G_8_ on both sides with A_−1_ T_0_|A_7_T_8_ resulted in a variant with clearly inferior BIL1/BZR1 binding (Fig. 1B). Substituting the C_3_G_4_ dinucleotides, characteristic of the G‐box motif, with other dinucleotides—A_3_G_4_, T_3_G_4_, G_3_G_4_, A_3_T_4_, T_3_A_4_, and G_3_C_4_—significantly reduced the binding affinity (Fig. 1B). Among these E‐box variants, C_1_A_2_ T_3_ G_4_T_5_G_6_ (complementary to C_1_A_2_C_3_ A_4_ T_5_G_6_) exhibited the highest affinity for the reduced BIL1/BZR1 DBD, maintaining slightly weaker binding capacity compared to the C_1_A_2_ or C_−1_A_0_ substitutions.

To further understand the binding specificity for the N_3_N_4_ bases, we carried out X‐ray crystallographic analysis of the reduced BIL1/BZR1 in complex with several DNA fragments containing the E‐box, C_−1_A_0_|C_1_A_2_N_3_N_4_T_5_G_6_|T_7_G_8_. Utilizing MBP as a crystallization chaperone in the same way as in the previous studies [9, 14, 21] (Table 1, Fig. 1C), we successfully resolved two complex structures of the reduced BIL1/BZR1 bound to DNA with E‐box variants T_3_A_4_ and G_3_C_4_. The three BIL1/BZR1–DNA complexes—each containing a DNA fragment with either the G‐box (C_3_G_4_) from the perfect BBS or two E‐box variants (T_3_A_4_ and G_3_C_4_)—exhibit highly similar conformations in both the entire protein‐DNA complexes and the extracted DNA fragments, with RMSDs around 0.2–0.3 Å (Fig. S1A,B). Apart from N_3_N_4_‐specific recognition, the protein‐DNA interactions were essentially identical among the complexes. In complex with the C_3_G_4_ bases, the guanidino groups of key Arg41 residues have rigidly formed hydrogen‐bond networks with O6 and N7 atoms of the G_4_ bases in both protomers [14] (Fig. 1D). However, with the E‐box containing T_3_A_4_, an Arg41 residue from one protomer recognized the N7 atom of the A_4_ base (protomer 1), while the Arg41 residue from the other protomer interacted with phosphate groups but not with the A_4_ base (protomer 2) (Fig. 1E). Guanine (G) and adenine (A), the two major purine bases, have different donor/acceptor properties, which possibly led to the distinguished hydrogen‐bond network robustness between the G_4_‐ and A_4_‐Arg41 interactions. In complex with the other E‐box harboring G_3_C_4_, the Arg41 residues of both protomers failed to directly recognize the C_4_ base, and no hydrogen‐bond‐mediating water molecules were observed at 2.2 Å resolution, potentially accounting for the weaker BIL1/BZR1 binding (Table 1, Fig. 1F). In addition, differences were also observed in the formation of water molecule‐mediated hydrogen bonds between the Glu37 residue and the A_2_ base (Fig. S1C). Although the structural basis for this difference remains unclear, BIL1/BZR1 is characterized by a relatively loose recognition of A_2_ via Glu37 [14, 21]. This may result from molecular fluctuations, as reflected in variations in *B*‐factors (Fig. S1A,B), possibly resulting from the weakened N_4_ recognition. Indeed, DNA fragments containing CA|CATATG|TG exhibited extremely weak competitive ability—that is, binding strength to BIL1/BZR1—comparable to those containing CA|CAGCTG|TG, with almost no detectable competition observed under our assay conditions. This suggests that the double‐base substitutions (C_3_G_4_ to T_3_A_4_ or G_3_C_4_) substantially impair binding, in contrast to the strong recognition observed for the canonical C_3_G_4_ in G‐box. By contrast, DNA fragments with single‐base substitutions, such as CA|CATGTG|TG and CA|CAGGTG|TG, showed clearly different levels of interaction with BIL1/BZR1, consistent with the differences in hydrogen bonding observed in the crystal structures of CA|CATATG|TG‐, CA|CAGCTG|TG‐harboring complexes (Fig. 1E, F). Overall, these findings provide structural insights into the DNA‐binding specificity for the N_3_N_4_ bases, which explains the binding ability of the reduced BIL1/BZR1 toward the E‐box variants.

### H_2_O_2_‐mediated oxidation impairs the DNA‐binding capability of BIL1/BZR1 through structural modifications

**Fig. 2:**
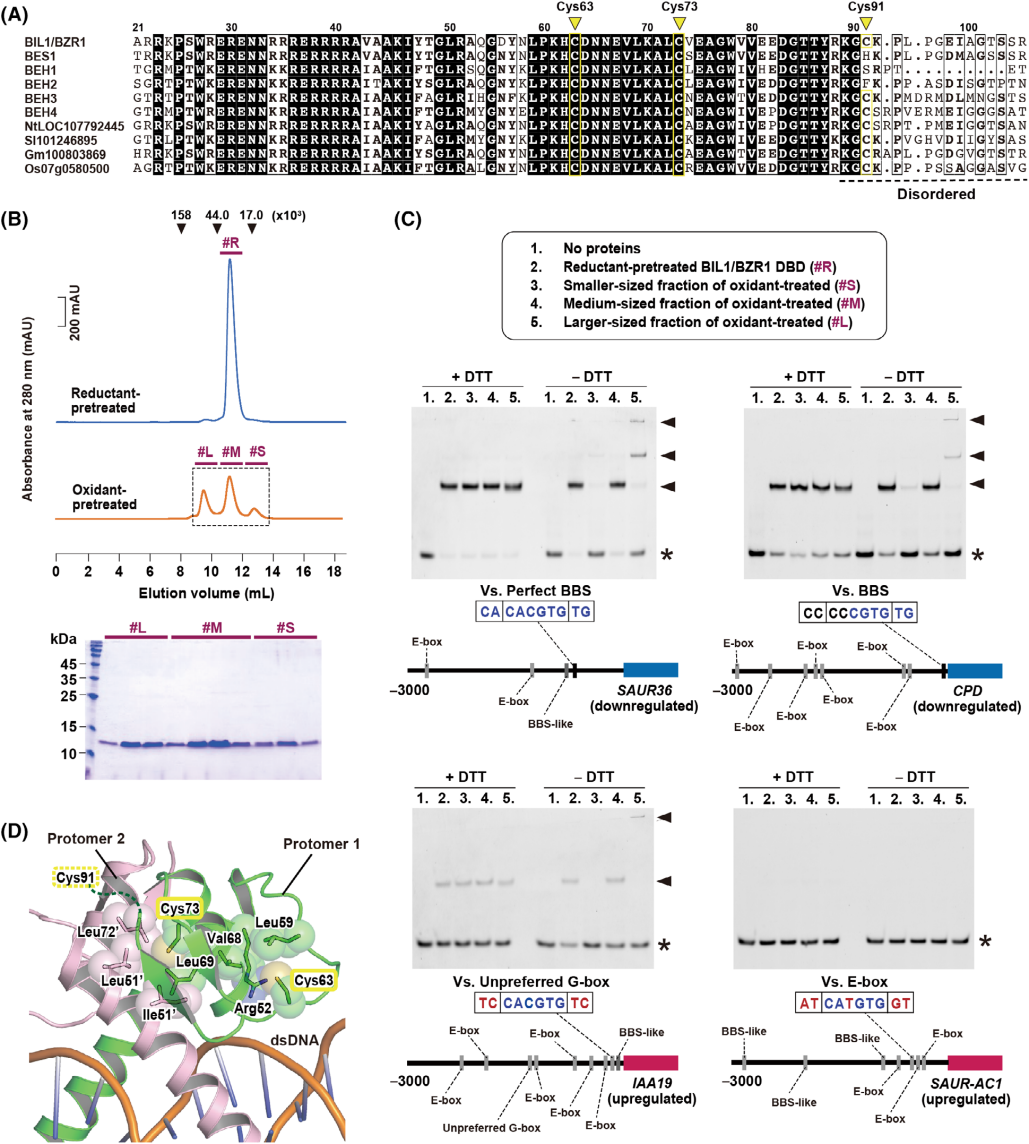
Oligomeric and DNA‐binding properties of the oxidized BIL1/BZR1 DBD. (A) An amino acid sequence alignment of BIL1/BZR1 homolog DBDs included the following proteins: *A. thaliana* BIL1/BZR1 (At1g75080), BES1 (At1g19350), BEH1 (At3g50750), BEH2 (At4g36780), BEH3 (At4g18890), and BEH4 (At1g78700); *Nicotiana tabacum* LOC107792445; *Solanum lycopersicum* 101 346 895; *Glycine max* 100 803 869; and *Oryza sativa* 07 g0580500. This alignment was performed using CLUSTAL OMEGA with default parameters, and the result was visualized using ESPript 3.0. The residues Cys63 and Cys73, which were completely conserved, along with the partially conserved Cys91, are highlighted with yellow inverted triangles. The disordered region where no electron density was observed in the previous crystal structure of BIL1/BZR1‐DBD in complex with DNA (PDB: 5ZD4) is shown at the bottom of the alignment. (B) SEC analyses were conducted on the unfused BIL1/BZR1 DBD after pretreatment with the reductant DTT and the oxidant H_2_O_2_, under nonreducing conditions. The upper panel displays the chromatogram, noting the peak positions of the marker proteins by their molecular masses. The lower panel shows a Coomassie‐stained gel image from SDS/PAGE analysis of the eluates from the oxidant‐pretreated samples. Fractions are labeled as follows: #R for the fraction pretreated with the reductant; #S, #M, and #L for smaller, medium, and larger‐sized fractions pretreated with the oxidant, respectively. (C) Gel shift assays using SEC‐separated BIL1/BZR1 DBDs pretreated with either reductant or oxidant. Assays included 4 μm of each protein and 0.25 μm of each FAM‐labeled DNA fragment from the promoters of various genes and revealed that following genes were differentially regulated under the BR‐response: *SAUR36* (downregulated), *CPD* (downregulated), *IAA19* (upregulated), and *SAUR‐AC1* (upregulated). These assays were conducted under both reducing (+DTT) and nonreducing (−DTT) conditions. The electrophoretic patterns are shown with asterisks (*) and triangular arrowheads (▲) indicating the position of the free FAM‐labeled DNA probe and unfused BIL1/BZR1‐DNA complex, respectively. The genomic locations of the *cis*‐elements in the extracted promoter regions are shown below each gel image. (D) The location of the conserved cysteine residues is mapped on the structure of the reduced BIL1/BZR1 DBD dimer complexed with DNA (PDB: 5ZD4). The two protomers of BIL1/BZR1 are colored differently. Amino acid residues involved in hydrophobic and van der Waals interactions, which stabilize the dimer and the loop architecture of the reduced BIL1/BZR1 DBD, are depicted in stick and sphere models. The beginning of the disordered region is marked by a dashed line.

Given the unclear differences in DNA‐binding specificity between the reduced and oxidized states, we assessed the biochemical properties of the oxidized BIL1/BZR1 DBD, which harbors the three cysteine residues Cys63, Cys73, and Cys91 (Fig. 2A). A previous study employing a biotinylated iodoacetamide (BIAM)‐labeling assay and biotin‐switch assay revealed that the Cys63 and Cys91 residues are major oxidized sites, and the highly conserved Cys63 residue is essential for canonical BR‐mediated responses [15]. Pre‐incubation of the unfused BIL1/BZR1 DBD with DTT as a reductant yielded a single peak in size exclusion chromatography analysis, indicating a uniform dimeric conformation as previously reported [31] (Fig. 2B, upper panel). In contrast, pretreatment with H_2_O_2_ as an oxidant resulted in three distinct peaks, suggesting multiple conformational states (Fig. 2B, upper panel). SDS/PAGE analysis under reducing conditions confirmed that these peaks comprised full‐length BIL1/BZR1 DBD (Fig. 2B, lower panel), indicating that the variations were due to conformational and/or oligomeric changes induced by H_2_O_2_ treatment.

We next evaluated the DNA‐binding properties of the oxidized BIL1/BZR1 DBD using four different DNA fragments from known target promoters [5, 6, 9, 10, 32, 33], each containing unique *cis*‐elements that variably bind the reduced BIL1/BZR1 DBD (Fig. 2C). Under the nonreducing reaction condition (−DTT), the medium‐sized fraction of oxidant‐treated BIL1/BZR1 (#M) showed almost the same DNA‐binding ability as the reductant‐pretreated fraction (#R), suggesting that this medium‐sized fraction remained in the reducing state (Fig. 2C). On the other hand, the smaller‐sized fraction of oxidant‐treated (#S) lost the DNA‐binding ability (Fig. 2C). Despite the upper band shift in the complex with DNA, the larger‐sized fraction (#L) also showed a decreased DNA‐binding ability compared to fractions #R and #M (Fig. 2C). Under the reducing reaction condition (+DTT), both fractions #S and #L regained sufficient DNA‐binding abilities, comparable to the levels of fractions #R and #M (Fig. 2C). Collectively, these results showed that H_2_O_2_‐mediated oxidation reversibly impairs the DNA‐binding capability of BIL1/BZR1 DBD, independent of the type of target nucleobases.

Based on the previously reported crystal structure of the BIL1/BZR1 DBD‐DNA complex [14, 21], the highly conserved Cys63 and Cys73 residues sustained the loop architecture and dimer interface of the DNA‐bound state, respectively (Fig. 2A,D). The partially conserved Cys91 residue was in a disordered region where no electron density was observed (Fig. 2A,D). Therefore, the oxidative modifications of Cys63 and Cys73—particularly Cys63, which has been identified as a primary oxidative target and a key residue involved in BR responses in a previous study—may predominantly disrupt the optimal dimeric architecture required for sufficient DNA binding, resulting in the decreased DNA‐binding ability.

### Neither the reduced nor the oxidized BIL1/BZR1 has heterodimerizing and/or cooperative DNA‐binding potential with PIF4 DBDs

**Fig. 3:**
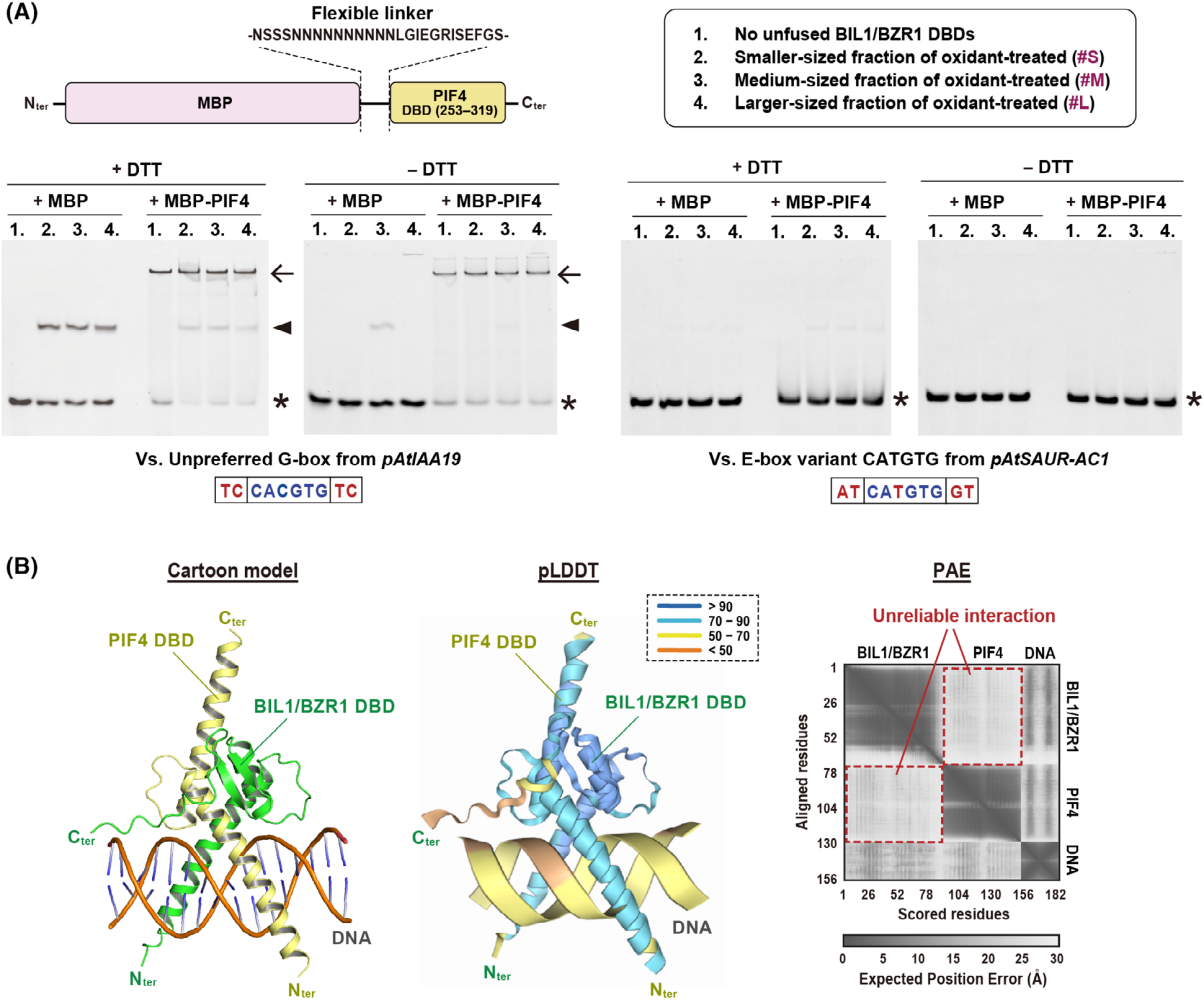
Analyses of heterodimerizing and cooperative DNA‐binding potential of BIL1/BZR1 and PIF4 DBDs. (A) Gel shift assays of the unfused BIL1/BZR1 DBD, pretreated with reductant or oxidant and separated by SEC, and the MBP‐fused PIF4 DBD connected via a flexible linker (the construct is shown in this panel). Assays included 4 μm of each protein and 0.25 μm of each FAM‐labeled DNA fragment from the promoters of *IAA19* and *SAUR‐AC1*. These assays were conducted under both reducing (+DTT) and nonreducing (−DTT) conditions. Electrophoretic patterns are shown with symbols indicating the positions of the free FAM‐labeled DNA probe (*), unfused BIL1/BZR1‐DNA complex (▲), and MBP‐fused PIF4‐DNA complex (←). (B) Results from AlphaFold3 prediction of the potential for heterodimerization between BIL1/BZR1 and PIF4 DBDs in complex with DNA harboring an unpreferred G‐box (TC|CACGTG|TC). The outputs included model confidence scores (pLDDT), predicted aligned error (PAE) values obtained from the AlphaFold server (https://alphafoldserver.com), and a cartoon model visualized using the PyMOL viewer of the top‐ranked structure.

The previous study suggested that oxidation of the cysteine residues within the DBD of BIL1/BZR1 enhances its interaction with PIF4 *in vivo*, leading to increased upregulation of BR‐induced genes [15]. This observation led us to hypothesize that the oxidized bHLH‐like DBD of BIL1/BZR1, with its disrupted homodimeric structure, might promote heterodimer formation with the bHLH DBD of PIF4. Consequently, we prepared an MBP‐fused PIF4 DBD connected via a flexible linker and conducted gel shift assays using two DNA fragments containing single *cis*‐elements (the unpreferred G‐box or the E‐box) enriched in the promoters of BR‐upregulated genes (Fig. 3A). However, all three fractions of the oxidant‐pretreated BIL1/BZR1 DBD failed to form heterodimers with the PIF4 DBD or to show cooperative increases in DNA binding to the prevalent *cis*‐elements, both under reducing and nonreducing conditions (with and without DTT, respectively) (Fig. 3A).

We additionally performed structure prediction of the DNA‐BZR1‐PIF4 ternary complex using AlphaFold3 [30]. The top‐ranked predicted ternary complex initially appeared to adopt a bHLH‐like heterodimeric structure, with relatively high pLDDT scores (> 70%) (Fig. 3B). However, PAE values, which reflect the uncertainty in the relative positioning of protein domains, were high between the BIL1/BZR1 and PIF4 DBDs, suggesting very low confidence in heterodimerization (Fig. 3B). Collectively, our gel shift assay and structural prediction suggest that the conformations of BIL1/BZR1 and PIF4 DBDs are not conducive to heterodimerization and/or cooperative DNA recognition.

## Discussion

In this study, we first showed that the preference of reduced BIL1/BZR1 for specific *cis‐*elements is governed by strong guanine‐specific interactions at the C_3_G_4_ bases in the 10‐bp BBS *cis‐*elements, which include the G‐box and BRRE motifs. Structural analyses indicated that the conserved Arg41 residue forms robust hydrogen bonds with the guanine base, which were diminished in other bases, thus explaining the reduced DNA‐binding affinity of BIL1/BZR1 toward the E‐box variants (C_1_A_2_N_3_N_4_T_5_G_6_, N_3_N_4_ ≠ C_3_G_4_). This is also consistent with the fact that there were almost no E‐box variants in the *A. thaliana*‐derived promoter fragment that strongly recruited BIL1/BZR1 in the previously reported Cistrome analysis [9, 34]. It was also found that CATGTG (complementary to CACATG) exhibited the highest affinity for the reduced BIL1/BZR1 among the E‐box variants, which may be related to its enrichment in BR‐induced upregulated genes.

**Fig. 4:**
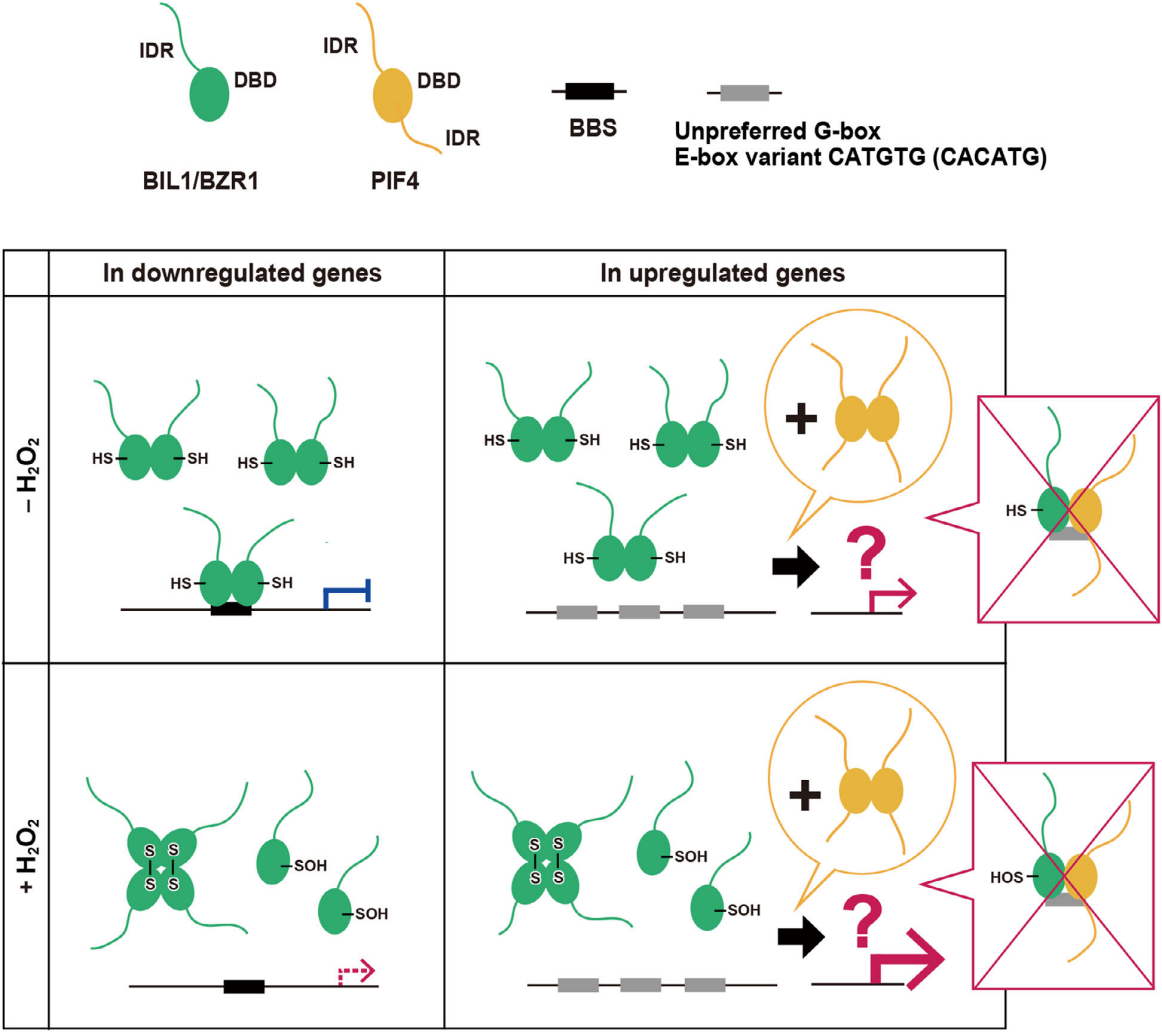
An updated integrative model of gene regulation by BIL1/BZR1 through H_2_O_2_‐mediated oxidation and interaction with PIF4. The reduced BIL1/BZR1 dimer preferentially binds to the BBS *cis*‐elements in their promoters. Upon H_2_O_2_‐mediated oxidation, the BIL1/BZR1 dimer conformation is disrupted, leading to a loss of strong DNA‐binding capability, which potentially relieves gene downregulation. In these BR‐induced downregulated genes, neither the reduced nor the oxidized forms of BIL1/BZR1 exhibit strong binding to single *cis‐*elements, such as the unpreferred G‐box and the E‐box variant CACATG, which are enriched in these genes. In addition, neither the reduced nor the oxidized BIL1/BZR1 DBDs demonstrate heterodimerizing or cooperative DNA‐binding potential with PIF4 DBDs. This regulatory process likely involves intrinsically disordered regions (IDRs) outside the DBDs, multiple *cis‐*elements, additional cofactors, and/or other indirect mechanisms.

Furthermore, the present findings deepen our understanding of the regulatory impact of oxidative modifications on the DNA‐binding capacity of BIL1/BZR1, independent of the type of target nucleobases. Oxidation of the conserved cysteine residues of BIL1/BZR1 may reversibly disrupt its structural features, such as the dimer interface and loop architecture, resulting in decreased DNA‐binding in various conformational states. Notably, the oxidized BIL1/BZR1 DBD failed to bind tightly even to the perfect BBS *cis*‐elements enriched in BR‐induced downregulated genes, including BR biosynthesis genes. This implies that the oxidative modification by the H_2_O_2_ signal may relieve feedback inhibition of BR signaling by the reduced BIL1/BZR1, potentially contributing indirectly to the activation of BR‐induced gene upregulation (Fig. 4).

Contrary to previous models, our gel shift assay demonstrated that the BIL1/BZR1 and PIF4 DBDs did not directly form heterodimers on *cis‐*elements, including the unpreferred G‐box and the E‐box variant CACATG, that are enriched in BR‐induced upregulated genes, even under oxidative conditions. AlphaFold3 predictions also supported the minimal interaction potential between these two proteins. Our study highlights the complexity of BR‐responsive transcriptional regulation, which cannot be fully explained by DNA‐binding specificity or redox regulation alone. While the cooperative actions of BIL1/BZR1 and PIF4 in gene regulation are evident, they likely involve the intrinsically disordered regions (IDRs) outside the DBDs, multiple *cis*‐elements, additional cofactors, and/or other indirect mechanisms (Fig. 4). Future research should focus on identifying these mediators to elucidate the molecular basis of BR‐induced upregulation and its integration with environmental and stress‐response pathways.

## Conflict of interest

The authors declare no conflict of interest.

## Author contributions

SN, TN, MT, and TM conceived and designed the research. SN and MO performed the biochemical experiments. SN prepared the crystals. SN and TM collected and processed the X‐ray diffraction data. SN wrote the initial draft of the paper. All authors reviewed the paper, and SN, MT, and TM edited the final paper.

## Supporting information


**Fig S1.** Structural comparison of reduced BIL1/BZR1‐DNA complexes.

## Data Availability

The atomic coordinates and structural factors of the mMBP‐fused BIL1/BZR1 in complex with the E‐box variants (CATATG and CAGCTG)‐containing DNA fragments have been deposited in the Protein Data Bank (PDB) under accession codes 9M73 and 9M74, respectively.
