# Supplementary material for: Single cis‐elements in brassinosteroid‐induced upregulated genes are insufficient to recruit both redox states of the BIL1/BZR1 DNA‐binding domain
Source: FEBS Lett. 2025 Aug 29;599(22):3369–80. doi: 10.1002/1873-3468.70147 (PMC12668046; doi:10.1002/1873-3468.70147)
Supplement: Supplementary file 1 — Fig S1. Structural comparison of reduced BIL1/BZR1‐DNA complexes. [file FEB2-599-3369-s001.docx]

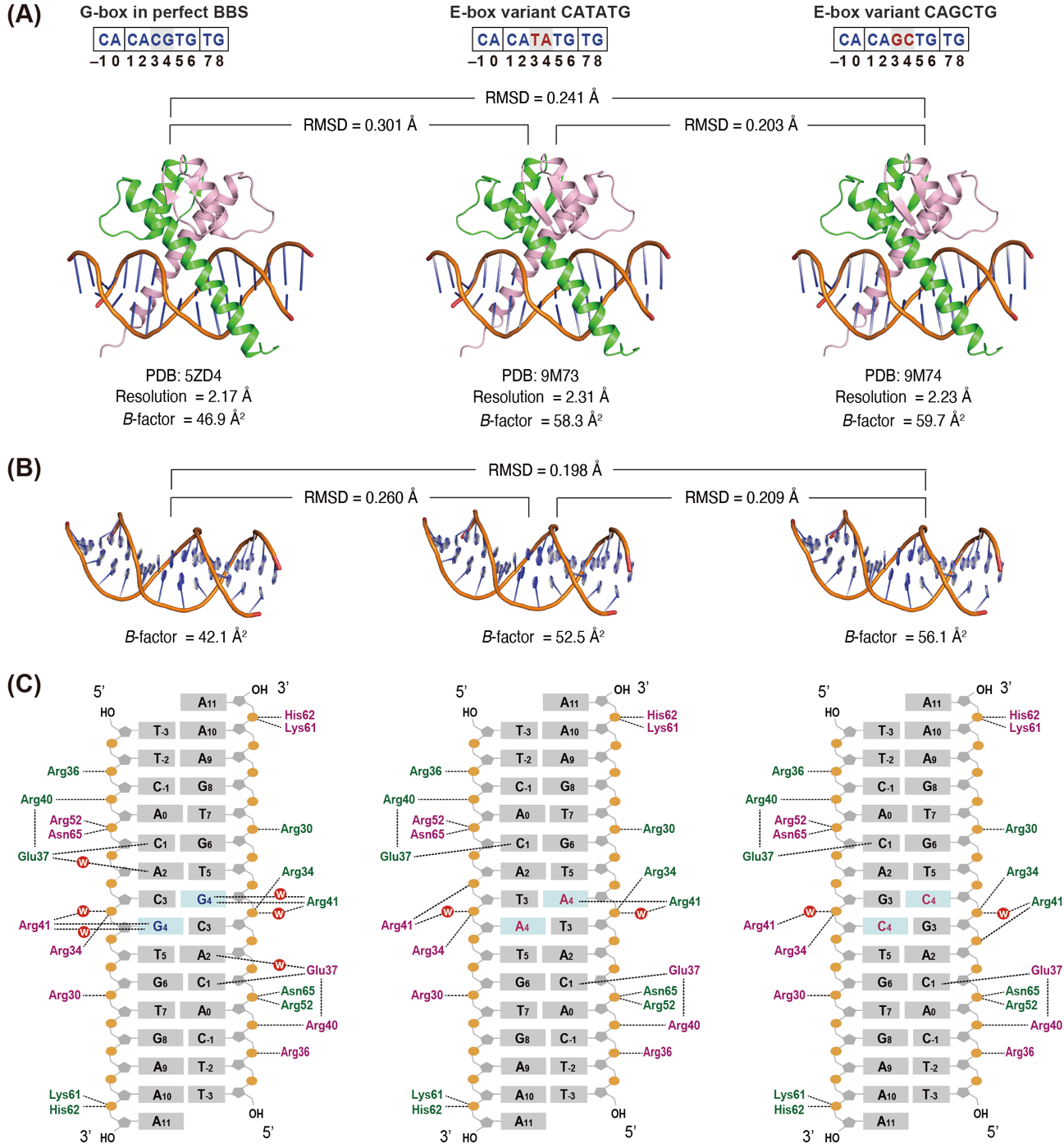


**Fig S1. Structural comparison of reduced BIL1/BZR1-DNA complexes.** (A) Structural comparison of the overall structure of three reduced BIL1/BZR1 in complex with DNA fragments, harboring the G-box motif in the perfect BBS (C_3_G_4_), and two variants of the E-box motif, T_3_A_4_ and G_3_C_4_. Each two BIL1/BZR1 protomers correspond to chains C and D in each PDB entry and belong to the assembly exhibiting higher overall *B*-factors. Overall *B*-factors and RMSD values among the complexes are also shown. (B) Structural comparison of the three DNA fragments extracted from the BIL1/BZR1-DNA complexes. Overall *B*-factors and RMSD values among the complexes are also shown. (C) Schematic models of the interactions between reduced BIL/BZR1 and three DNA fragments. Hydrogen-bonding and salt-bridge interactions are indicated by black dashed lines. Each N_4_ base is highlighted with a cyan background. Amino acid residues from different BIL1/BZR1 protomers are shown in two distinct colors. Water molecules mediating hydrogen bonding are represented as red spheres labeled with “W.” DNA phosphate groups are shown as orange circles.
